# Response to: Correcting for cell-type effects in DNA methylation studies: reference-based method outperforms latent variable approaches in empirical studies

**DOI:** 10.1186/s13059-017-1149-7

**Published:** 2017-01-30

**Authors:** Kevin McGregor, Aurélie Labbe, Celia M. T. Greenwood

**Affiliations:** 1 Lady Davis Institute for Medical Research, Jewish General Hospital, Montreal, QC Canada; 2 Department of Epidemiology, Biostatistics and Occupational Health, McGill University, Montreal, QC Canada; 3 Departments of Oncology and Human Genetics, McGill University, Montreal, QC Canada; 4 Department of Decision Sciences, HEC Montreal, Montreal, QC Canada

## Abstract

We thank Hattab and colleagues for their correspondence and their investigation of cell-type mixture correction methods in methyl-CG binding domain sequencing. Here, we speculate on why surrogate variable analysis (SVA) performed differently between their two data sets, and poorly in one of them.

Please see related Correspondence article: https://genomebiology.biomedcentral.com/articles/10/1186/s13059-017-1148-8 and related Research article: https://genomebiology.biomedcentral.com/articles/10.1186/s13059-016-0935-y

## Response

We enjoyed reading the recent correspondence by Hattab et al. [1] on recommendations when adjusting for cell-type mixtures in methyl-CG binding domain sequencing (MBD-seq). Hattab and colleagues discussed their concern about the performance of SVA in their analyses. We note, importantly, that all our simulations were based on the methylation profiles obtained from the Illumina Infinium HumanMethylation450K BeadChip and not on data arising from a sequencing-based platform. This could be responsible for important differences in performance as SVA is a linear method, and the characteristics of sequencing data—such as variable read depth, discreteness of methylation estimates, and the number of zeros that tend to occur—could impact performance; this would be worth investigating. We do concede that, for datasets with a very large number of features, such as MBD-seq, reference-free methods are rather impractical owing to computational complexity.

In our simulations, SVA was not always the best-performing method among those methods that do not require external cell-type-specific reference methylation profiles. However, SVA seemed to be the safest choice as it never failed badly and its performance was close to the best method for each simulation scenario. In the correspondence by Hattab et al., the difference between the two datasets in terms of the performance of SVA is intriguing. The study design and the quality-control approaches for the schizophrenia study were well described [2] and included employing a random order for sample processing as well as careful curation of misaligned reads. It would be interesting to know whether, or how, these steps differ from those undertaken in the more recent depression study and whether the characteristics of read depth and coverage differed between the two data sets, which could lead back to our concern about using a linear method for count-derived measures of methylation.

We also have some reservations with respect to the measure of performance reported by Hattab et al. [1]. Enrichment of detected sites makes the inherent assumption that there are at least some true positive sites to be detected. When evaluating performance in real data, although we agree that it is impossible to know the truth, this particular metric is not ideal if there are no true positives to be found. It would be interesting to investigate how SVA and the reference-based methods compare if performance were to be assessed by empirical false discovery rate estimates, such as those described elsewhere [3].

Finally, we are in firm agreement with Hattab and colleagues that the best results are likely to be achieved with the reference-based method, using appropriate reference methylation profiles, and that obtaining such reference methylation profiles is worth the effort whenever possible. However, there are some tissues and cell types where this is extremely difficult, if not impossible (e.g., syncytiotrophoblasts in placenta), and, in such situations, alternative cell-type mixture correction methods will still be needed.

## Abbreviations

MBD-seq: Methyl-CG binding domain sequencing
SVA: Surrogate variable analysis
